# The Effects of Group Therapeutic Singing on Cortisol and Motor Symptoms in Persons With Parkinson's Disease

**DOI:** 10.3389/fnhum.2021.703382

**Published:** 2021-07-26

**Authors:** Elizabeth L. Stegemöller, Andrew Zaman, Mack Shelley, Bhavana Patel, Ahmad El Kouzi, Elizabeth A. Shirtcliff

**Affiliations:** ^1^Department of Kinesiology, Iowa State University, Ames, IA, United States; ^2^Department of Statistics, Iowa State University, Ames, IA, United States; ^3^Department of Political Science, Iowa State University, Ames, IA, United States; ^4^Department of Neurology, University of Florida, Gainesville, FL, United States; ^5^Department of Neurology, Southern Illinois University School of Medicine, Springfield, IL, United States; ^6^Department of Human Development and Family Studies, Iowa State University, Ames, IA, United States

**Keywords:** singing, cortisol, Parkinson's disease, motor symptoms, UPDRS

## Abstract

The inclusion of music into the treatment plan for persons with Parkinson's disease (PD) may be a viable strategy to target multiple motor symptoms. However, potential mechanisms to explain why music has an impact on multiple motor symptoms in persons with PD remain understudied. The purpose of this study was to examine the acute effects of 1 h of group therapeutic singing (GTS) on physiological measures of stress and clinical motor symptoms in persons with PD. We posit that improvement in motor symptoms after GTS may be related to stress reduction. Seventeen participants with PD completed 1 h of GTS and eight participants completed 1 h of a quiet reading (control session). Cortisol was collected via passive drool immediately before and after the singing and control session. The Unified Parkinson's Disease Rating Scale (UPDRS) Part-III (motor examination) was also video-recorded immediately before and after the singing and control session and scored by two raters masked to time and condition. Secondary outcome measures for quality of life, depression, and mood were collected. Results revealed no significant change in cortisol or motor UPDRS scores, as well as no significant relationship between cortisol and motor UPDRS scores. There was a trend for the singing group to report feeling less sad compared to the control group after the 1-h session (effect size = 0.86), and heart rate increased in the singing group while heart rate decreased in the control group after the 1-h session. These results suggest that an acute session of GTS is not unduly stressful and promotes the use of GTS for persons with PD. Multiple mechanisms may underlie the benefits of GTS for persons with PD. Further exploring potential mechanisms by which singing improves motor symptoms in persons with PD will provide greater insight on the therapeutic use of music for persons with PD.

## Introduction

Over the next 20 years, the prevalence of Parkinson's disease (PD) is likely to double. Yet, there is no cure. Current predominant forms of treatment (i.e., drug therapy and deep brain stimulation) provide substantial relief but have significant side effects and are expensive (Borgohain et al., 2012; Borovac, 2016), and many symptoms of PD are not fully ameliorated by current treatments. Thus, there is a pressing need to develop therapeutic strategies that limit side effects, reduce treatment costs, and target multiple symptoms of PD.

Dance and music have been incorporated into current treatment strategies for PD. Learning and performing ballroom dance steps improved functional mobility, gait, and postural stability (Hackney and Earhart, 2009, 2010; Foster et al., 2013). Ballet and Irish dancing have resulted in acute improvements in functional mobility and postural instability (Houston and McGill, 2013; Volpe et al., 2013). Drumming has been shown to improve walking rate in persons with PD (Pantelyat et al., 2016). Our group has shown that group therapeutic singing (GTS) improved respiratory control, swallow, and quality of life (Stegemöller et al., 2016, 2017a). The singing groups were enjoyable for participants as they offered a way to relieve stress and have fun (Stegemöller et al., 2017b). Participants also viewed the groups as an avenue to express their concerns about having PD and build camaraderie with other people with PD (Stegemöller et al., 2017b). Taken together, this suggests that the inclusion of music into the treatment plan for persons with PD may be a viable strategy to target multiple motor symptoms as well as improve mood and quality of life.

Building on evidence showing promising outcomes, the next step is to uncover potential mechanisms to explain why music has an impact on multiple motor symptoms in persons with PD. The positive benefits of dance and drumming in PD may be related to increased physical activity as prior studies have shown that participation is associated with improvements in balance, gait, risk for falls, physical function, sleep cognition, and quality of life (Feng et al., 2020). However, it remains challenging to isolate the benefits of music vs. physical activity. GTS does not require an overt amount of physical activity, yet various motor symptoms and quality of life are improved. Further exploring the mechanism by which singing improves motor symptoms in persons with PD will provide greater insight on the therapeutic effects of music alone.

Building from our previous research revealing that participants with PD reported feeling less stressed after group therapeutic singing (Stegemöller et al., 2017b) and research that demonstrated music and singing can reduce perceived stress and reduce cortisol in various clinical populations (Miluk-Kolasa et al., 1994; Scheufele, 2000; Khalaf et al., 2003; Fukui and Toyoshima, 2008; Bradt et al., 2014; Fancourt et al., 2016), we posit that the improvement in motor symptoms may be due to reduced stress. When persons with PD experience stress, their motor symptoms frequently worsen (van der Heide et al., 2021). Indeed, when clinically evaluating tremor, patients are often given a mild cognitive stressor (i.e., count backwards by three) to trigger the emergence of tremor. To our knowledge, no study has examined the effects of singing on stress (or stress reduction) in persons with PD. The purpose of this study is to determine the acute effects of group therapeutic singing on clinical motor symptoms and stress. We hypothesized that after 1 h of singing, (1) clinical motor scores would improve, (2) cortisol, a biomarker of stress, would decrease, and (3) there would be a relationship between improved clinical motor scores and cortisol.

## Methods

### Participants

Twenty-five participants were enrolled into the study. Inclusion criteria included age between 40 and 85, a diagnosis of PD, and on the same PD medication for the past 30 days. Exclusion criteria included a score <24 on the Mini Mental State Exam. Demographic. Disease information at the day of study enrollment is shown in Table 1. All participants were tested on their optimal PD medication, following their regular timing and dosage, as prescribed by their treating physician. Time since last PD medication is shown in Table 1. All participants gave written informed consent prior to inclusion in the study, and the Institutional Review Board of Iowa State University approved the procedures. Participants were recruited from ongoing GTS groups in surrounding areas as well as from a general listserve of persons with PD interested in research. Participants currently participating in a GTS group were assigned to the singing intervention group. Those participant not currently participating in a GTS group were assigned to the control group. This resulted in 17 participants enrolled in the singing session, and eight participants enrolled in the control session.

**Table 1:** Participant demographic and disease information.

**Participant**	**Gender**	**Age**	**DD (years)**	**MMSE**	**BDI**	**PDQ-39**	**Total UPDRS**	**Singing (years)**	**Medication (minutes)**
1	F	78	5	29	23	58	28	2	150
2	F	80	5	29	12	64	39	3	150
3	F	74	6	30	10	30	29	0.25	300
4	F	84	8	30	10	50	26	0.5	120
5	M	70	2	30	10	33	31	2	180
6	M	79	7	26	13	57	40	0.17	150
7	M	85	1	26	8	14	19	0.75	180
8	F	64	2	30	12	46	29	2	330
9	M	73	12	30	6	47	32	3	430
10	F	73	13	30	8	35	34	4	210
11	M	83	7	24	14	76	40	3	210
12	F	69	16	30	14	27	27	4	180
13	M	67	4	30	9	46	42	2	210
14	F	77	17	29	8	69	38	1	300
15	F	69	6	28	24	85	40	4	120
16	F	77	6	28	22	66	45	4	240
17	F	61	11	29	12	42	28	4	45
18*	M	77	4	30	12	15	20	0	135
19*	M	72	1	26	12	69	37	0	330
20*	F	66	6	29	5	53	34	0	60
21*	M	74	15	27	5	61	42	0	270
22*	M	68	2	30	3	11	17	0	210
23*	M	67	8	30	13	30	32	0	240
24*	F	65	8	30	3	6	12	0	150
25*	F	71	6	30	7	23	14	0	120
Mean ± SD (Singing group)	NA	74.29 ± 1.70	7.50 ± 1.15	28.76 ± 0.40	12.65 ± 1.32	49.71 ± 4.57	33.35 ± 1.71	2.37 ± 0.33	206.18 ± 22.41
Mean ± SD (Control group)	NA	70.00 ± 1.48	6.25 ± 1.54	29.00 ± 0.57	7.50 ± 1.49	33.50 ± 8.58	26.00 ± 4.08	0	189.38 ± 31.43
*t* score, *p*-value, Hedges *g* (*df* = 23)	NA	1.59, 0.12, 0.68	0.64, 0.53, 0.28	−0.34, 0.74, 0.14	2.36, 0.03, 1.01	1.83, 0.80, 0.78	1.63, 0.12, 0.46	NA	0.23, 0.82, 0.22

*Asterisks designate participants in the control group. Shaded cells indicate significant differences between groups. Medication is time in minutes since last taking their normal PD medication to the start of the singing session. SD, Standard Deviation; DD, Disease Duration; MMSE, Mini Mental State Exam; BDI, Beck Depression Inventory; PDQ-39, Parkinson's Disease Quality of Life Scale; UPDRS, Unified Parkinson's Disease Rating Scale; Singing, Number of Years Singing; Medication, Time Since Parkinson's Medication*.

### Singing Session

Seventeen participants completed 1 h of GTS. The singing session began with a greeting song lasting ~5 min. A series of vocal exercises lasting ~15 min followed the greeting song and included diaphragmatic breathing exercises, lip buzzing, glissandos, and articulation exercises. The vocal exercises have been used in previous group therapeutic singing studies.^11−13^ Specific songs targeting pitch range, articulation, and breath support followed the vocal exercises for ~15 min. Participants were then asked to choose songs they would like to sing for ~20 min. The session concluded with a closing song lasting ~5 min. Participants completed all songs and vocal exercises without written music or lyrics. A review of lyrics was provided as needed prior to singing each song. Participants were instructed to sit with appropriate posture, breathe from the diaphragm, lift the palate, and show facial expression while singing. A piano was used to accompany the vocal exercises and songs. The session was led by a board certified music therapist with over 15 years' experience leading therapeutic singing groups for persons with PD.

### Control Session

Eight participants completed 1 h of a control (i.e., no singing) session. Participants were instructed to sit in a quiet room together and read quietly for 1 h. The room was in the same building where the singing sessions were held.

### Data Collection

Data collection for both groups were completed using the same location. Prior to completing the singing or control session, participants completed a series of questionnaires. The Parkinson's Disease Questionnaire (PDQ-39) was collected as a measure of quality of life and the Beck Depression Index (BDI) was collected as a measure of depression (Jenkinson et al., 1997; Goodarzi et al., 2016). The total Unified Parkinson's Disease Rating Scale (MDS-UPDRS) was collected as a measure of disease severity (Martinez-Martin et al., 2013). Number of years singing in the therapeutic singing group was also collected (Table 1). In addition, a daily diary was completed documenting food intake, exercise, tobacco use, and unusual events for 24 h prior to data collection. The daily diary also included a subjective report of anxiety, anger, happiness, and sadness on a scale from 1 to 7. This scale was completed prior to and after the singing or control session and served as secondary outcome measures for further exploratory analyses (Table 2). Resting heart rate and blood pressure collected pre and post-session are shown in Table 2.

**Table 2:** Change scores for subjective mood ratings, heart rate, and blood pressure.

**Participants**	**Anxiety**	**Anger**	**Happiness**	**Sadness**	**Heart rate**	**Systolic BP**	**Diastolic BP**
1	1	1	5	−2	24	0	−2
2	0	0	0	0	18	33	8
3	0	0	0	0	−2	0	−1
4	0	0	0	−1	20	0	9
5	−1	0	2	−3	−4	8	0
6	0	0	−1	−1	6	10	10
7	−1	0	4	−1	−2	5	5
8	−1	−1	0	−2	0	2	−3
9	1	0	−2	0	−2	−11	8
10	−3	0	−2	−1	−8	−4	−18
11	1	0	1	−1	0	3	38
12	−2	0	0	−1	22	−2	−8
13	−3	0	2	0	−8	1	2
14	0	0	−3	0	14	26	−4
15	0	0	2	0	−4	14	−2
16	−1	0	3	−1	−2	21	2
17	−1	0	1	0	−10	−7	−1
18*	0	0	0	0	−1	3	−3
19*	3	1	−3	−1	−1	−14	15
20*	−1	0	−2	0	−7	7	18
21*	−4.5	0	6	1	4	19	6
22*	0	0	0	0	−21	15	5
23*	−1	0	3	−1	−4	50	30
24*	−1	0	0	0	−4	−3	8
25*	−3	0	0	0	−15	−6	6
Mean ± SD (Singing group)	−0.59 ± 0.30	0.00 ± 0.09	0.71 ± 0.52	−0.82 ± 0.21	3.65 ± 2.76	5.82 ± 2.86	2.53 ± 2.78
Mean ± SD (Control group)	−0.94 ± 0.78	0.12 ± 0.12	0.50 ± 1.00	−0.12 ± 0.23	−6.12 ± 2.88	8.75 ± 7.03	10.62 ± 3.58
*t* score, *p*-value, Hedges *g* (*df* = 23)	0.51, 0.61, 0.22	−0.82, 0.42, 0.35	0.20, 0.84, 0.3	−1.99, 0.06, 0.86	2.17, 0.04, 0.93	−0.46, 0.65, 0.20	−1.71, 0.10, 0.73

*Asterisks designate participants in the control group. Shaded cells indicate significant differences between groups. SD, Standard Deviation; BP, Blood Pressure*.

### Unified Parkinson's Disease Rating Scale

The motor MDS-UPDRS was used as the primary outcome measure for clinical motor symptoms. The scale was administered by a trained rater immediately before and after the singing session. Video recordings were completed and later scored by two movement disorders neurologists that were masked to the study. The neurologists were not informed of the intervention used (i.e., singing or control), and the videos were coded to mask pre or post-intervention order.

### Cortisol

The primary outcome measure of cortisol, a widely used marker of stress, was collected immediately before and after the singing session via passive drool into sterile Wheaton Cryogenic 2 ml vials. The sessions were held from 3 to 4 p.m. central standard time, so the sample was collected within 30 min before and 30 min after the sessions. Because salivating can be challenging for patients with PD, we provided participants with water 5 min prior to sample collection, and participants were taught to use a chewing motion to enhance flow. To limit potential stress of providing a sample, participants were instructed that they had 5 min to complete the sampling and that whatever they were able to produce was sufficient. For cortisol, only 50 μL of saliva is needed for duplicate tests, which most participants provided within 1–2 min. Samples were frozen within 1 h at −80°C for later analysis.

Cortisol was analyzed with the Salimetrics® Cortisol Enzyme Immunoassay Kit, a commercially-available FDA cleared kit with a wide detection range (0.007 ug/dL−3 ug/dL) and minimal cross-reactivity with other biomarkers. Saliva was assayed in duplicate. Duplicates that varied by >7% were re-assayed. With each assay plate, a standard curve was calculated; standard curves that were *R* < 0.997 were re-assayed. High and low controls were also calculated with each assay plate; controls that are out of range or varied by >20% were re-assayed. Duplicates were averaged, reported as ug/dL, and inspected for normality. Typical for cortisol, the distribution was skewed, so the data were winsorized and log-transformed.

### Statistical Analysis

Normality was tested using the Shapiro-Wilk test. Data followed approximately a normal distribution, including cortisol which was transformed. Independent-sample *t*-tests were conducted for the two sets of motor UPDRS scores to determine if there were differences between the masked raters' scores. No significant differences were revealed (pre-session scores: *p* = 0.48; post-session scores: *p* = 0.55). Therefore, the average of the two motor UPDRS scores was calculated and used for the remaining statistical analyses. Independent-samples *t*-tests probed for differences between groups for the demographic data, disease information data, and change scores (post-value–pre-value) for the secondary outcome measures, heart rate, and blood pressure. Effect sizes using Hedges' *g* were calculated, as sample sizes were different between groups.

To test the hypothesis that clinical motor scores will improve and cortisol will decrease after an acute session of singing, a 2 (pre- and post-session scores) × 2 (singing vs. control group) repeated measures ANOVA was estimated for each primary outcome measure (motor UPDRS and cortisol). Any demographic or disease variable that was found to be significantly different between groups was entered as a covariate in the ANOVA. *Post-hoc* analyses were completed using Tukey's Honestly Significant Difference test. Significance was set at *p* < 0.05. To determine if there was a difference in the magnitude of change in the primary outcome measures (motor UPDRS and cortisol) between groups, independent-samples *t*-tests were conducted using the change scores (post-value–pre-value).

To test the hypothesis that there was a relationship between clinical motor scores and cortisol, a Pearson product-moment correlation was estimated to determine the relationship between motor UPDRS and cortisol across both groups, and for the singing group and control group separately. Change scores were calculated (post-value–pre-value) and used as data for the Pearson correlation.

Additional exploratory analyses were completed to determine if demographics, disease information, or change in secondary outcome measures (i.e., subjective reports of anxiety, anger, happiness, and sadness) account for the change in motor UPDRS scores or cortisol. A stepwise linear regression model was used to determine the predictors of the change in each primary outcome measure (motor UPDRS and cortisol) for both groups combined and for each group separately (singing and control). Using change scores for the primary outcome measures, participants were categorized based on their response (decrease or increase/no change). A chi-square test for differences in the percentage of participants in each group (singing vs. control) demonstrated a decrease in motor UPDRS scores. A parallel separate chi-square test for difference in the percentage of participants in each group demonstrated a decrease in cortisol. Significance was set a *p* < 0.05. Effect sizes using Cramer's *V* were calculated for the chi-square tests.

## Results

Data from the daily diary indicated that all participants had eaten a meal within 3 h before the first data collection, nine of the participants (five in the singing group, four in the control group) had exercised on the day of the data collection (all ~5 h prior), no participants used tobacco, and there were no significant unusual events within 24 h prior to the first data collection. Comparisons between groups for demographic and disease information revealed a significant difference for only the BDI [*t*_(23)_ = 2.36, *p* = 0.03, *g* = 1.01]. The singing group had a higher score than the control group. No other comparisons attained statistical significance (Table 1). For blood pressure and heart rate, results revealed a significant difference between groups for heart rate only [*t*_(23)_ = 2.17, *p* = 0.04, *g* = 0.93]. Heart rate increased after the session for the singing group while heart rate decreased after the session for the control group (Table 2). For the secondary outcome measures (participant reports of mood) there were no significant differences between groups, although there was a trend for the singing group to report feeling less sad than the control group [*t*_(23)_ = −1.99, *p* = 0.06, *g* = 0.86] (Table 2).

### Motor UPDRS and Cortisol

**Figure 1:**
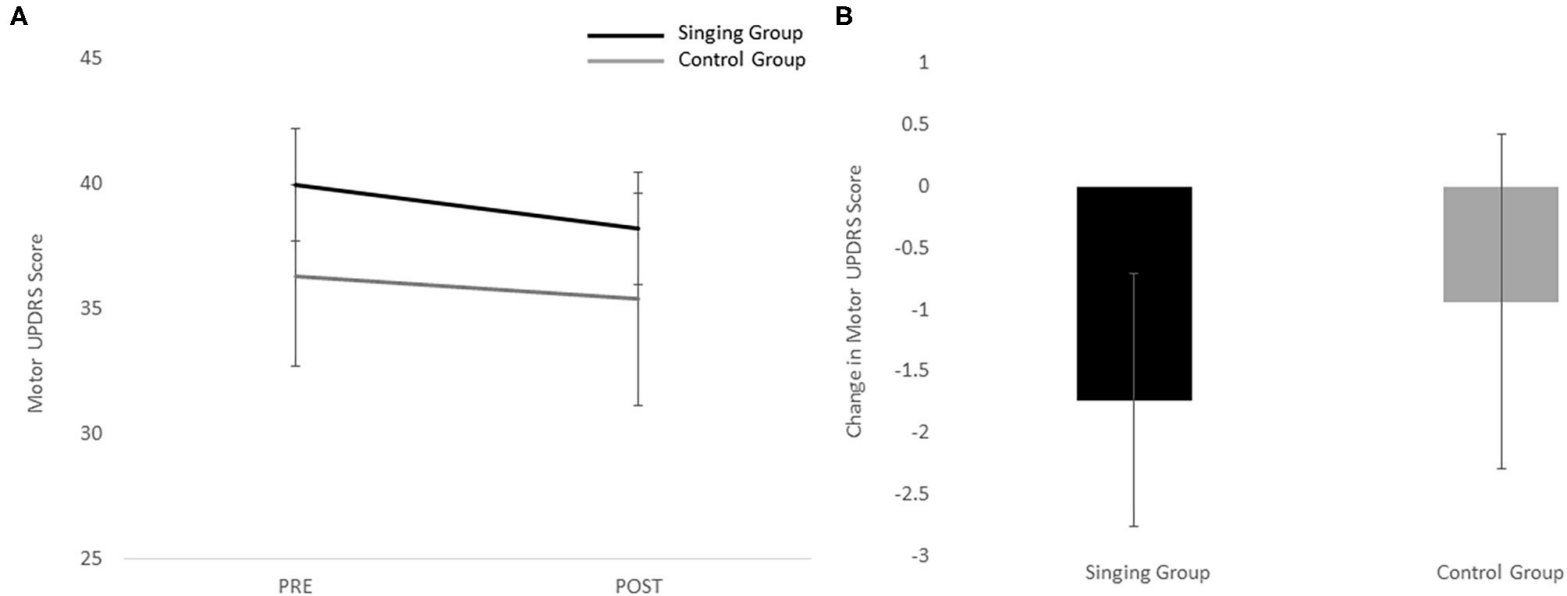
**(A)** Pre and post-scores for the singing and control group for the motor UPDRS. **(B)** Change scores for the motor UPDRS for both the singing and control groups.

**Figure 2:**
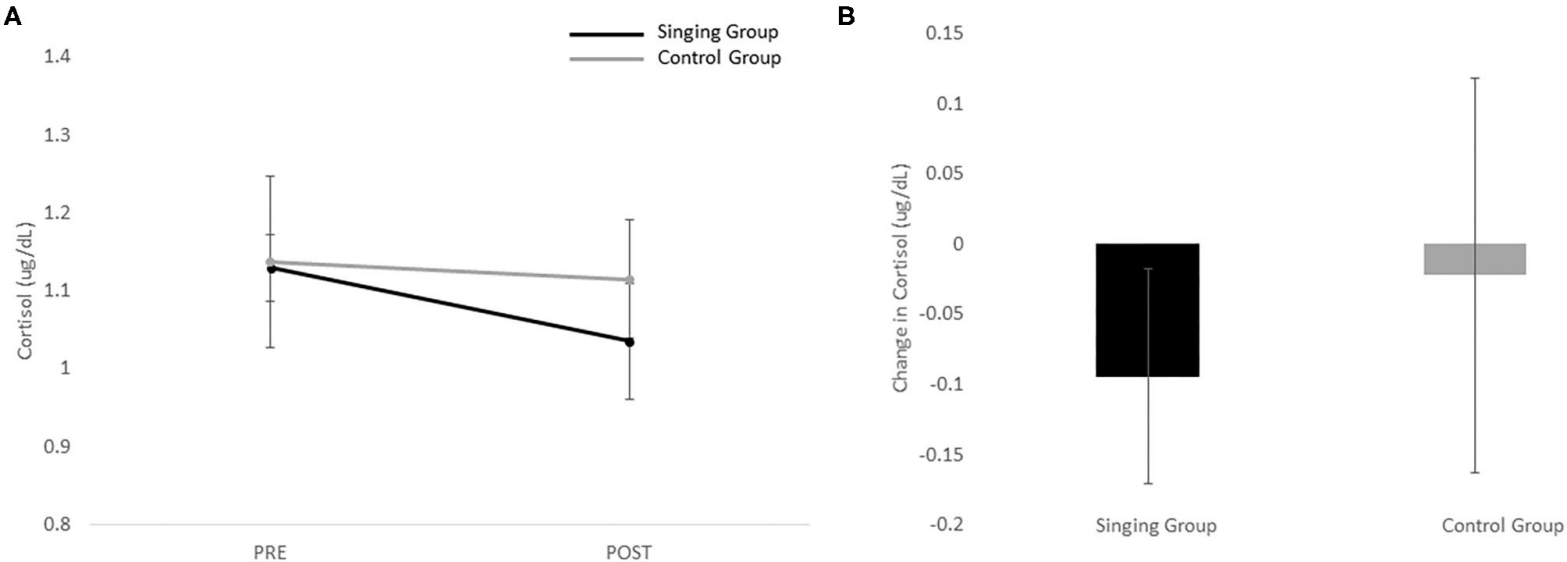
**(A)** Pre and post-values for the singing and control group for cortisol. **(B)** Change in cortisol for both the singing and control groups.

Individual pre and post-scores for the motor UPDRS and cortical are shown in Table 3. Because there was a significant difference between groups, the BDI was entered as a covariate in the repeated measures ANOVA for the motor UPDRS and cortisol. In general, the singing group demonstrated a greater decrease in motor UPDRS scores (Figure 1) and cortisol (Figure 2) than the control group, but no significant differences were revealed. Results revealed no main effect of session [*F*_(1)_ = 1.43, *p* = 0.24], no main effect of group [*F*_(1)_ = 0.34, *p* = 0.57], and no interaction effect [*F*_(1)_ = 0.21, *p* = 0.65] for the motor UPDRS. Similarly, results revealed no main effect of session [*F*_(1)_ = 1.38, *p* = 0.25], no main effect of group [*F*_(1)_ = 0.95, *p* = 0.34], and no interaction effect [*F*_(1)_ = 0.24, *p* = 0.63] for cortisol. Comparisons of change scores also revealed no significant differences between groups for both motor UPDRS [*t*_(23)_ = −0.45, *p* = 0.65, *g* = 0.19] and cortisol [*t*_(23)_ = −0.49, *p* = 0.63, *g* = 0.21]. Finally, no significant associations between motor UPDRS and cortisol were revealed for both groups combined (*R* = 0.20, *p* = 0.34), the singing group only (*R* = 0.05, *p* = 0.83), nor the control group (*R* = 0.47, *p* = 0.24).

**Table 3:** Individual participant pre and post-scores for the motor UPDRS and cortisol.

**Participants**	**Pre-motor UPDRS**	**Post-motor UPDRS**	**Pre-cortisol (ug/dL)**	**Post-cortisol (ug/dL)**
1	29	26	1.05	1.04
2	47	48.5	1.40	0.75
3	41	37	1.16	1.02
4	35.5	37.5	1.40	0.74
5	42	32	1.05	0.85
6	48	51	1.06	1.23
7	33	30	0.96	1.33
8	29	32.5	1.24	1.20
9	48	44.5	1.40	1.56
10	52.5	41	0.81	0.73
11	49	47.5	0.86	0.89
12	36	33.5	1.25	1.36
13	36	37.5	1.11	1.45
14	57	57.5	1.22	1.12
15	34.5	37	1.02	1.31
16	38.5	35.5	1.01	0.41
17	23	21	1.19	0.80
18*	37	37	1.28	0.84
19*	48.5	50	0.86	0.92
20*	20	22.5	0.71	1.32
21*	51.5	54	0.93	1.45
22*	32	23	1.28	1.12
23*	38	38	1.49	1.20
24*	28	24.5	0.98	0.90
25*	35.5	34	1.56	1.17

*Asterisks designate participants in the control group*.

### Exploratory Analyses

Stepwise linear regression revealed that only the PDQ-39 was a significant predictor of the change in motor UPDRS scores when both groups were combined (*t* = 2.7, *p* = 0.01, *B* = 0.09). No significant predictors were revealed for the change in cortisol when both groups were combined. For the singing group only, there were no significant predictors for the change in motor UPDRS or in cortisol. Similarly for the control group only there were no significant predictors for the change in motor UPDRS or in cortisol. Finally, there were no differences in the number of participants who demonstrated a decrease in the motor UPDRS [*X*_2_(1) = 0.99, *p* = 0.32, *V* = 0.19] or a decrease in cortisol [*X*_2_(1) = 0.03, *p* = 0.86, *V* = 0.03] between the singing and control groups.

## Discussion

The purpose of this study was to determine the effects of GTS on clinical motor symptoms and stress. We hypothesized that clinical motor scores would improve, cortisol would decrease, and there would be a relationship between clinical motor scores and cortisol. Results revealed that clinical motor symptoms improved and cortisol decreased in some, but not all, persons with PD, and no relationship between clinical motor scores and cortisol was revealed. To better understand other factors that might contribute to the null results, exploratory analyses were completed. Of interest, there was a trend for the singing group, which had a significantly higher BDI score, to feel less sad compared to the control group after the 1-h session (effect size = 0.86), and heart rate increased in the singing group while heart rate decreased in the control group after the 1-h session. These results suggest that an acute session of GTS is not stressful and other mechanisms may underlie the benefits of group therapeutic singing for persons with PD.

### Singing as a Stressor

Previous research has shown that singing can reduce cortisol acutely in some populations (Fancourt et al., 2016). Based upon previous subjective reports that participants with PD felt less stressed after GTS (Stegemöller et al., 2017b), we hypothesized that cortisol would decrease after 1 h of GTS. Only 10 of the 17 participants (59%) in the singing group demonstrated a decrease in cortisol after the singing session while five of the eight participants (62%) in the control group demonstrated a decrease in cortisol after the control session. Thus, it is equally plausible that group singing could be stressful (Reschke-Hernández et al., 2017; Everaerd et al., 2020). Previous research has shown that elements such as performance, an audience/group, and stepping outside of one's comfort zone can increase stress (von Dawans et al., 2011; Shirtcliff et al., 2014; Smyth et al., 2015). All of these elements are involved in singing. In addition, persons with PD often have voice impairments, which may also contribute to an increase in stress when singing due to perceptions of self-consciousness and/or embarrassment (Lewis and Ramsay, 2002; Gruenewald et al., 2004).

While the results did not show a significant decrease in cortisol as hypothesized, results also did not show a significant increase in cortisol. Participants also reported a decrease in sadness and anxiety with an increase in happiness after GTS. This may suggest that the therapeutic nature of GTS may have mediated the potential stress associated with singing. Indeed, research has shown that cortisol reactivity is diminished when all the elements of stress are removed (Het et al., 2009; Wiemers et al., 2013). Results also revealed that heart rate increased in the GTS group and decreased in the control group, which is characteristic of a stress response (Thayer et al., 2012). However, prior studies have found that heart rate also changes dramatically with deep breathing, which is a focus in GTS. This raises the question of whether the increase in heart rate we observed during GTS is due to stress, engagement, or deep breathing. We speculate that singing induced a mild stress response commensurate with being engaged with the session (Seery, 2011). Moreover, the lack of cortisol response suggests that the increase in heart rate was not due to unmanageable levels of stress or challenge. This speculation is bolstered by the reduced motor symptoms in some participants, which typically abate during relaxation and worsen during stress. Nonetheless, these findings support the notion that a single session of GTS is not unduly stressful which is a positive finding and promotes the use of GTS for persons with PD.

### Underlying Mechanisms of GTS

The change in motor UPDRS scores reported in this study was variable across participants. In 10 of the 17 participants who completed GTS, the motor UPDRS score decreased an average of four points. Moreover, in two of these participants the motor UPDRS score decreased by more than 10 points. These changes in scores occur on average 3.5 h after taking medication. Thus, other mechanisms outside of medication possibly mediate these improvements. We hypothesized that the change in motor UPDRS score would be related to cortisol, and that a reduction in stress would be an underlying mechanism. During stress in healthy older adults, the top-down pathways involved in executive functioning, explicit learning, and performing novel skills are impaired (Petersen et al., 1998; Poldrack et al., 2005; Foerde and Shohamy, 2011). Persons with PD often use top-down pathways to compensate for the loss of more automatic processes mediated by the basal ganglia. Since stress impairs top-down processes, persons with PD must rely more on the impaired basal ganglia pathway, resulting in greater movement impairment. Thus, if singing reduced stress, then movement impairment would be less. However, results did not show a significant association between reduction in motor UPDRS score and cortisol. It is possible that the influences on motor symptoms may take longer than one session and require long-term drops in cortisol. Indeed, slow stable shifts in cortisol basal activity is considered a more adaptive profile whereas large declines or rises may indicate an allostatic stress state (Epel et al., 1998; Korte et al., 2005; Lupien et al., 2006). Future studies evaluating the long-term changes in cortisol are warranted to further understand the underlying mechanisms associated with improved motor symptoms with GTS.

Other mechanisms may underlie the previously reported benefits of GTS. Results revealed that there was a trend for the singing group, which had a significantly higher BDI score, to feel less sad compared to the control group after the 1-h session (effect size = 0.86). Thus, changes in mood, specifically depression, may account for improvements in motor UPDRS scores. Neuroimaging studies have revealed that listening to music stimulates dopaminergic regions, including the nucleus accumbens and ventral tegmental area (VTA) (Menon and Levitin, 2005; Koelsch et al., 2006; Salimpoor et al., 2011; Chanda and Levitin, 2013). These regions play a role in regulating emotion, specifically depression. Moreover, the VTA also has dopaminergic projections to the primary motor cortex (M1) that directly modulate the excitability of M1 neurons (Luft and Schwarz, 2009; Kunori et al., 2014). Thus, modulation of the VTA may underlie improvements in both mood and motor UPDRS scores in persons with PD after GTS.

Results of this study do not directly confirm the involvement of either proposed mechanism, and it is likely that the variability of response to GTS may be at play. Specifically, the relation between arousal and/or stress and movement performance may explain the variable results. Yerkes and Dodson (1908) first described this relationship as an inverted U-curve, where movement performance is best at a moderate level of arousal and movement performance suffers at extreme levels (high or low) of arousal (Yerkes and Dodson, 1908). This relationship is thought to be mediated by the locus coeruleus (Berridge and Waterhouse, 2003; Aston-Jones and Cohen, 2005; Aston-Jones and Waterhouse, 2016) which is part of the stress system (Godoy et al., 2018). While research is limited in how this applies to motor symptoms in persons with PD, there is clinical evidence that stress exacerbates motor symptoms. Thus, the variable relationship between cortisol and motor symptoms after GTS revealed in this study may be explained by the inverted-U principle. Some participants may be above the optimal zone (elevated arousal due to anxiety and stress) while other participants may be below the optimal zone (low arousal or drowsy). Indeed, fatigue and day-time drowsiness is a very common non-motor symptom for persons with PD. Moreover, GTS may modulate stress through engagement, which may combat low arousal or decrease stress through deep breathing, which may combat elevated arousal or anxiety. The variability in the results of this study may be due to multiple factors that are influencing cortisol and its potential impact on motor symptoms. Further studies are needed aimed at delineating the magnitude of stress, challenge, or stress release during GTS, and how this relates to motor symptoms in persons with PD.

### Limitations

Many of the outcome measures did not attain statistical significance but had decent effect sizes, and we acknowledge the study was underpowered. Nonetheless, the results provide the initial steps in better understanding the benefits of GTS for persons with PD. While food intake was recorded, participants were not required to limit intake prior to the study. Some participants had food within an hour so a post-prandial change in cortisol cannot be ruled out. Finally, the GTS and control groups were not matched. A study design that controls for demographic and disease information is needed. However, significant differences between groups were limited, which still allows for the interpretation of results in light of the small differences between groups. While participants in this study reported a diagnosis of idiopathic PD, it is difficult to confirm. Atypical PD has a different trajectory and can impact motor symptoms differently. Thus, there is the possibility that participants with atypical PD may be included in the sample, though their current diagnosis at time of data collection was idiopathic PD, and could have affected the results.

## Conclusion

This study is among the first to examine the acute effects of GTS on physiological markers of stress and the relationship to motor symptoms in persons with PD. Results revealed that there was no significant change in cortisol after 1 h of GTS, providing the initial evidence that GTS is not stressful in the tested participants with PD. Moreover, the response to GTS was variable across the participant sample, suggesting that further studies are needed to understand better both how participant and intervention aspects modulate stress. Nonetheless, this study provides the initial evidence that GTS is not stressful, and in some persons with PD, GTS can improve motor symptoms.

## Data Availability Statement

The raw data supporting the conclusions of this article will be made available by the authors, without undue reservation.

## Ethics Statement

The studies involving human participants were reviewed and approved by Iowa State University Institutional Review Board. The patients/participants provided their written informed consent to participate in this study.

## Author Contributions

ESt: writing of first draft, data collection, data analysis, and participant recruitment. ESh: writing of first draft and data analysis. AK and BP: editing of manuscript and UPDRS rating. MS: editing of manuscript and statistical analysis. AZ: editing of manuscript, data collection, and data analysis. All authors contributed to the article and approved the submitted version.

## Conflict of Interest

The authors declare that the research was conducted in the absence of any commercial or financial relationships that could be construed as a potential conflict of interest.

## Publisher's Note

All claims expressed in this article are solely those of the authors and do not necessarily represent those of their affiliated organizations, or those of the publisher, the editors and the reviewers. Any product that may be evaluated in this article, or claim that may be made by its manufacturer, is not guaranteed or endorsed by the publisher.
